# Exploring the Relationship Between Neutrophil-to-Lymphocyte Ratio and Glycemic Control in Type 2 Diabetes Mellitus: A Cross-Sectional Analysis

**DOI:** 10.3390/jcm15051844

**Published:** 2026-02-28

**Authors:** Gulam Saidunnisa Begum, Elham Said Ahmed AlRisi, Salima Al-Maqbali, Vijaya Marakala, Geetha Subramaniam, Anshoo Agarwal, Nida Suhail, Mariah N. Hafiz, Mohammed M. Jawad

**Affiliations:** 1 Department of Biochemistry, College of Medicine and Health Sciences, Sohar Campus, National University of Science and Technology, Sohar 321, Oman; gulambeguam@nu.edu.om (G.S.B.); vijayamarakala@nu.edu.om (V.M.); 2 Department of Pathology and Blood Bank Sohar Hospital, Ministry of Health, Sohar 321, Oman; elhamalrisi@yahoo.com (E.S.A.A.); almoq96@yahoo.com (S.A.-M.); 3 Faculty of Health and Life Sciences, INTI International University, Nilai 71800, Negeri Sembilan, Malaysia; geetha.subramaniam@newinti.edu.my; 4 Department of Pathology, Faculty of Medicine, Northern Border University, Arar 91431, Saudi Arabia; dranshoo3@gmail.com; 5 Department of Medical Laboratory Technology, Faculty of Applied Medical Sciences, Northern Border University, Arar 91431, Saudi Arabia; mariah.hafiz@nbu.edu.sa (M.N.H.); mohammed.jawad@nbu.edu.sa (M.M.J.)

**Keywords:** type 2 diabetes mellitus, neutrophil-to-lymphocyte ratio, glycemic control, systemic inflammation, inflammatory biomarker, HbA1_C_

## Abstract

**Background/Objectives:** The neutrophil-to-lymphocyte ratio (NLR) is an emerging inflammatory biomarker associated with various metabolic disorders, including type 2 diabetes mellitus (T2DM). While poor glycemic control is linked to increased risk of complications, the relationship between glycemic status and NLR remains insufficiently characterized. This study aimed to assess the association between NLR and glycemic control in T2DM patients by comparing those with good glycemic control (GGC; HbA1c ≤ 7%) and poor glycemic control (PGC; HbA1c ≥ 7.1%). **Methods:** A retrospective cross-sectional study was conducted on 2907 T2DM patients from a tertiary care center. Patients were categorized into GGC (n = 1500) and PGC (n = 1407) groups based on HbA1c values. The mean age of participants was 53.53 ± 18.86 years in the GGC group and 54.31 ± 18.40 years in the PGC group, with comparable sex distribution between groups. Data were retrieved from electronic medical records and NLR was calculated using complete blood counts. Descriptive statistics were computed, and group comparisons were performed using Mann–Whitney U and independent *t*-tests. Spearman correlation and receiver operating characteristic (ROC) analysis were also performed. A *p*-value < 0.05 indicated significance. **Results:** The PGC group exhibited significantly higher mean NLR (3.96 ± 4.53) compared to the GGC group (3.23 ± 3.80) (*p* < 0.001). Median and interquartile range values confirmed this difference, and both parametric and non-parametric analyses supported the findings. Spearman correlation demonstrated a weak but statistically significant association between HbA1c and NLR (r = 0.101, *p* < 0.001). ROC analysis showed limited discriminatory ability of NLR (AUC = 0.560). Exploratory CRP results further suggested a link between inflammation and impaired glycemic control. **Conclusions:** NLR was significantly elevated in T2DM patients with moderate-to-poor glycemic control. This suggests that NLR may serve as a simple, cost-effective, and accessible marker of systemic inflammation and glycemic status. However, the effect size was small, and its discriminatory ability was limited, indicating that NLR should be interpreted as a supportive marker rather than a standalone clinical tool.

## 1. Introduction

Diabetes mellitus (DM) is a complex metabolic disorder affecting the regulation of blood glucose [1]. It primarily includes two types based on insulin secretion: type 1 DM (T1DM) and type 2 DM (T2DM) [2]. T2DM, often associated with immune system dysfunction, is increasingly recognized as an inflammatory disease [3]. Low-grade chronic inflammation plays a critical role in the development of T2DM, especially by promoting insulin resistance linked to obesity [4]. Elevated leukocyte counts reflect this persistent inflammation, which contributes to both macrovascular and microvascular diabetic complications [5,6]. Additionally, T2DM is associated with changes in serum inflammatory markers, including mean platelet volume (MPV) and cytokine levels [7,8]. Poor glycemic control, indicated by elevated HbA1c, promotes chronic inflammation and oxidative stress, leading to increased neutrophil-to-lymphocyte ratio (NLR) [9]. Elevated NLR has been linked to a higher risk of cardiovascular events, diabetic complications, and mortality in patients with diabetes. Alongside HbA1c, NLR may provide complementary information regarding inflammatory status offer additional prognostic value in assessing the risk of complications and mortality in T2DM patients. High HbA1c correlates with adverse outcomes [10] and is widely used in clinical monitoring [11]. However, HbA1c does not capture ongoing inflammatory processes and can be influenced by various factors such as genetic conditions, hematologic disorders, and illnesses [12,13].

NLR is recognized as a valuable marker of inflammation in both cardiac and non-cardiac conditions, offering superior predictive and diagnostic power compared to total leukocyte counts [14,15,16]. Normal NLR in adults’ ranges between 1 and 2, with values > 3.0 or <0.7 considered pathological. Intermediate values (2.3–3.0) may signal early pathological processes, including cancer, atherosclerosis, infections, inflammation, psychiatric disorders, or stress [17].

Elevated NLR levels have been reported in several inflammatory conditions, including type 2 diabetes mellitus, cancer, inflammatory bowel disease, cardiac disorders, thyroiditis, and COVID-19 infection [18,19,20,21,22,23,24]. NLR is increasingly recognized as a simple and accessible marker of systemic inflammation and an independent predictor of morbidity and mortality [25,26]. It reflects the balance between inflammatory activation and immune regulation, with neutrophilia and relative lymphopenia contributing to elevated values [27,28,29,30,31]. Compared to individual leukocyte counts, NLR is considered a more stable and cost-effective biomarker with reduced physiological variability [32,33,34].

Because NLR is derived from routine complete blood count testing, it represents an accessible and cost-effective marker that may be useful in clinical monitoring.

The association between diabetes mellitus and the neutrophil-to-lymphocyte ratio has emerged as a significant area of investigation [35]. NLR has demonstrated predictive utility comparable to established inflammatory biomarkers, including C-reactive protein (CRP), tumor necrosis factor-alpha (TNF-α), and interleukin-6 (IL-6), particularly in the detection of subclinical inflammation and endothelial dysfunction [36]. However, data evaluating the relationship between NLR and glycemic control in individuals with type 2 diabetes mellitus in Oman remain limited. Therefore, the present study aimed to evaluate NLR in individuals with T2DM and compare NLR levels between those with good glycemic control (HbA1c ≤ 7%) and poor glycemic control (HbA1c > 7.1%) to assess its potential role as an inflammatory marker in this population.

## 2. Materials and Methods

A retrospective cross-sectional observational study was conducted by reviewing the medical records of individuals treated at Suhar Hospital, Oman, between March 2023 and April 2024. Data on demographics, HbA1c, and complete blood counts (CBCs) were extracted from the electronic medical records using a structured Excel-based data collection form. CRP available data were analyzed as an exploratory marker. AI assistance was used solely for language editing. All statistical analyses were performed independently by the authors using their original dataset.

### 2.1. Ethical Approval and Consent to Participate

This study was approved by the college ethics and biosafety committee (NU/COMHS/EBC007/2024) and the Ministry of Health Research and Ethical Review Committee, North Batinah Governorate, Oman (RERAC-NBG: MH/DGHS/NBG:MoH/CSR/24/29179). The requirement for consent to participate was waived by the Ethics Committee due to the retrospective nature of the study and the use of anonymized, de-identified patient data. Therefore, individual informed consent was not obtained.

### 2.2. Sample Size

All eligible patients during the study period (n = 2907) were included. A post hoc power analysis was performed using the observed mean difference in NLR between groups, assuming a two-sided alpha of 0.05 and power of 80%, to confirm that the available sample size was adequate to detect statistically significant differences.

### 2.3. Study Design

Patients were categorized into two groups based on HbA1c levels, good glycemic control (GGC; HbA1c ≤ 7%) and poor glycemic control (PGC; HbA1c > 7.1%), using the established clinical guideline cutoff of 7%, which is recommended as the target for good glycemic control [2]. NLR was calculated by dividing the absolute neutrophil count by the absolute lymphocyte count, as recorded in the CBC results.

### 2.4. Inclusion and Exclusion Criteria

The study included adults (≥18 years) with type 2 diabetes mellitus (T2DM) who had documented HbA1c and complete blood count (CBC) available in their electronic medical records. Comorbidity data was not consistently available and were therefore not included in the analysis. Patients were excluded if they had other forms of diabetes (e.g., type 1 or gestational diabetes), pregnancy, lactation, acute infection, inflammatory or hematological disorders, malignancy, autoimmune disease, corticosteroid or immunosuppressant therapy. These conditions were identified and excluded based on documented clinical diagnoses, physician notes, medication history, and laboratory records available in the electronic medical record system at the time of data extraction.

### 2.5. Statistical Analysis

Data were coded in Microsoft Excel and analyzed using SPSS Version 21. Descriptive statistics were used to summarize the data. Baseline demographic characteristics including age and sex were summarized and compared between glycemic groups. Continuous variables, such as the Neutrophil–Lymphocyte Ratio (NLR), were assessed for normality using appropriate tests and were found to be non-normally distributed. Therefore, they were expressed as median and interquartile range (IQR), and group comparisons between individuals with good glycemic control (GGC) and poor glycemic control (PGC) were primarily performed using the Mann–Whitney U test. However, given the large sample size, independent samples *t*-test with Welch correction was also performed as a supplementary analysis, as parametric tests are considered robust to non-normality in large samples. A *p*-value of <0.05 was considered statistically significant. CRP available data were analyzed as an exploratory marker. CRP measurements were included only when available in the medical records and obtained during the same clinical assessment period as HbA1c and CBC. The proportion of missing CRP data was documented and excluded from CRP-specific analyses.

## 3. Results

The study included a total of 2907 individuals with type 2 diabetes, comprising 1500 with good glycemic control (GGC; HbA1c ≤ 7%) and 1407 with poor glycemic control (PGC; HbA1c > 7.1%). The demographic and clinical characteristics of the study population are presented in Table 1. The mean age was comparable between individuals with good glycemic control (53.53 ± 18.86 years) and poor glycemic control (54.31 ± 18.40 years). The sex distribution was also similar between groups, indicating that the groups were broadly comparable.

The mean Neutrophil–Lymphocyte Ratio (NLR) was 3.23 (SD = 3.80) in the GGC group and 3.96 (SD = 4.54) in the PGC group, showing a statistically significant difference (*p* < 0.001) (Table 2). Post hoc power analysis indicated that a minimum of 2064 participants would be required to detect this difference with 80% power at a 0.05 significance level. Given the study sample size of 2907 participants, the statistical power was adequate. The calculated Cohen’s d was 0.175, indicating a small effect size. Similarly, CRP levels were significantly higher in individuals with poor glycemic control compared to those with good glycemic control (*p* < 0.001).

Levene’s test indicated unequal variances between the glycemic groups for NLR (F = 26.20, *p* < 0.001). Therefore, Welch’s *t*-test was used for comparison. The mean NLR was significantly higher in the PGC group compared to the GGC group, with a mean difference of 0.73 units (*p* < 0.001), and the 95% confidence interval (0.43 to 1.03) did not include zero (Table 3).

Similarly, CRP levels were significantly higher in the PGC group, with a mean difference of 21.41 units (*p* < 0.001) and a 95% confidence interval of 16.27 to 26.55 (Table 3).

The percentile distribution of NLR is presented in Table 4. The median NLR was higher in the PGC group (2.28) compared to the GGC group (1.93). Similarly, the 75th percentile was higher in the PGC group (4.71) than in the GGC group (3.74).

CRP percentiles also showed higher values in the PGC group across all percentile levels (Table 4).

On performing the Mann–Whitney U test, a statistically significant difference in NLR levels was observed between individuals with poor glycemic control (PGC) and those with good glycemic control (GGC) (*p* < 0.001). Similar results were obtained using independent samples *t*-test as a supplementary analysis (Table 2).

Spearman correlation analysis demonstrated a weak but statistically significant positive correlation between HbA1c and NLR (r = 0.101, *p* < 0.001), indicating that higher HbA1c levels were associated with slightly higher NLR values.

ROC analysis showed that the AUC for NLR was 0.560 (95% CI: 0.539–0.580, *p* < 0.001) and for CRP was 0.593 (95% CI: 0.573–0.614, *p* < 0.001) (Table 5). Both markers demonstrated weak discriminatory ability in distinguishing poor from good glycemic groups, with CRP showing slightly higher AUC than NLR.

## 4. Discussion

This cross-sectional study aimed to assess the association between the Neutrophil–Lymphocyte Ratio (NLR) and glycemic control in individuals with type 2 diabetes mellitus (T2DM), categorized into two groups based on HbA1c: good glycemic control (GGC; HbA1c ≤ 7%) and poor glycemic control (PGC; HbA1c > 7.1%).

The demographic characteristics were comparable between the glycemic groups, with similar mean age and sex distribution. This reduces the likelihood that differences in NLR were due to major demographic imbalances between groups.

The mean NLR was significantly (*p* < 0.001) higher in the PGC group compared to the GGC group. The clinical utility of NLR may lie in its accessibility, trend monitoring, and potential adjunctive role alongside other biomarkers rather than as a strong individual predictor. The median NLR values were also higher in the PGC group compared to the GGC group, indicating a consistent trend of elevated systemic inflammation in patients with suboptimal glycemic control. The elevated NLR in PGC individuals supports the growing evidence that chronic low-grade inflammation plays a central role in the pathogenesis and progression of T2DM and its complications. As NLR reflects the balance between innate (neutrophil-driven) and adaptive (lymphocyte-mediated) immune responses, its elevation could indicate immune dysregulation associated with poor glycemic control. While the study does not establish a causal relationship, the consistent statistical significance across both parametric and non-parametric tests strengthens the observed association between NLR, exploratory marker CRP and HbA1c-defined glycemic control. However, the observed effect size was small (Cohen’s d = 0.175), indicating that the magnitude of difference between groups was modest.

Correlation analysis demonstrated a weak but statistically significant positive association between HbA1c and NLR (r = 0.101, *p* < 0.001), indicating that higher HbA1c levels were associated with slightly elevated NLR values. However, the strength of this correlation was weak, suggesting limited clinical relevance. This finding supports the interpretation that, although NLR reflects systemic inflammation, its utility as an independent marker of glycemic control is limited and should be interpreted with caution.

The magnitude of correlation observed in our study is consistent with previous reports demonstrating weak to modest associations between inflammatory markers and glycemic parameters. Hussain et al. reported a positive but modest correlation between NLR and HbA1c, while the meta-analysis by Adane et al. also showed variability in correlation strength across studies. These findings suggest that, although NLR is associated with glycemic status, the relationship is generally weak and likely reflects broader inflammatory processes rather than serving as a reliable independent indicator of glycemic control.

Our findings are also consistent with those of Mohammed et al., who reported significantly higher NLR values in patients with T2DM compared to non-diabetic individuals (4.189  ±  4.154 vs. 4.095  ±  8.851, *p* = 0.009), suggesting the potential role of NLR in reflecting systemic inflammation and aiding in diabetes management. Although their sample was smaller and showed high variability, their results reinforce the observed association between elevated NLR and poor glycemic status [37]. Similar findings were reported by Hussain et al., who investigated the association between NLR and levels of glycemic control in 330 T2DM patients. Their study demonstrated that NLR levels were significantly higher in patients with poor glycemic control and positively correlated with HbA1c values although the strength of correlation was modest [38]. These findings support our observation that NLR increases with worsening glycemic control, highlighting its potential role as an accessible inflammatory biomarker in the management of T2DM.

The findings of this study are further reinforced by a systematic review and meta-analysis conducted by Adane et al., which included 13 studies assessing the association between NLR and glycemic control in individuals with type 2 diabetes mellitus. The analysis demonstrated a consistent correlation between elevated NLR and higher HbA1c levels, concluding that NLR could serve as a supportive marker of glycemic control in T2DM patients [16]. This aligns with our results, where significantly elevated NLR values were observed in individuals with poor glycemic control compared to those with good control.

Our research confirms a significant association between higher NLR values and poor glycemic control in individuals with type 2 diabetes mellitus (T2DM), suggesting that NLR could serve as an accessible and cost-effective supportive inflammatory marker in clinical practice, rather than a standalone diagnostic tool. However, ROC analysis demonstrated limited discriminatory ability (AUC = 0.560), further supporting that NLR should be considered a supportive rather than a definitive clinical marker. Our study further highlights the potential utility of the Neutrophil–Lymphocyte Ratio (NLR) not only as a marker of glycemic control but also as a potential supportive indicator, although its predictive value remains limited.

Despite the statistical significance observed in this study, the magnitude of association between NLR and glycemic control was modest, as reflected by the small effect size, weak correlation coefficient, and limited discriminatory ability. This suggests that, while NLR reflects systemic inflammation, its ability to distinguish between levels of glycemic control is limited and may have restricted clinical applicability when used alone. Furthermore, the possibility of residual confounding cannot be excluded, as several important clinical variables such as duration of diabetes, medication use, and comorbid conditions were not available for adjustment. Therefore, these findings should be interpreted cautiously and viewed as supportive evidence rather than definitive proof of a strong clinical association.

This study has several limitations. As a retrospective, cross-sectional analysis, causality between NLR and glycemic control cannot be established. In addition, the analysis was based primarily on unadjusted comparisons between glycemic control groups, and multivariable adjustment was not performed due to lack of complete data on important clinical confounders such as duration of diabetes, body mass index, smoking status, medication use, and comorbidities. Therefore, the independent association between NLR and glycemic control cannot be confirmed. Furthermore, other factors such as concurrent infections and use of anti-inflammatory medications may have influenced NLR levels. These limitations may have affected the observed associations, and future prospective studies incorporating comprehensive clinical variables and multivariable regression analysis are required to validate these findings.

However, as this was a retrospective study, exclusion was based on available medical records, and the possibility of misclassification due to incomplete documentation cannot be entirely excluded.

## 5. Conclusions

This cross-sectional study demonstrates a statistically significant elevation in the Neutrophil–Lymphocyte Ratio (NLR) among individuals with poor glycemic control (HbA1c > 7.1%) compared to those with good glycemic control (HbA1c ≤ 7%) in patients with type 2 diabetes mellitus. Although the observed difference in NLR was modest (mean difference ≈ 0.73; Cohen’s d = 0.175), the finding supports the association between suboptimal glycemic control and increased systemic inflammation. A weak but statistically significant correlation between HbA1c and NLR further supports this association, although the strength of relationship was limited. Given its affordability, accessibility, and derivation from routine complete blood count tests, NLR may serve as a practical supportive marker in monitoring glycemic control—especially in resource-constrained settings. However, due to its small effect size and limited discriminatory power (AUC = 0.560), NLR should not be used as a standalone marker for clinical decision-making. Future prospective studies incorporating multivariable adjustment and comparison with established inflammatory biomarkers are required to further evaluate the independent association and potential supportive role of NLR, rather than its use as a definitive clinical marker, in diabetes management.

## Figures and Tables

**Table 1:** Demographic and clinical characteristics of study participants.

Variable	Good Glycemic Control (n = 1500)	Poor Glycemic Control (n = 1407)
Age (years), mean ± SD	53.53 ± 18.86	54.31 ± 18.40
Male, n (%)	669 (44.6%)	677 (48.1%)
Female, n (%)	831 (55.4%)	730 (51.9%)

**Table 2:** Comparing NLR (primary outcome) and CRP (exploratory marker) between individuals with good and poor glycemic control.

Group Statistics					
	DM	N	Mean	Standard Deviation	*p* Value
NLR	Good Control (GGC)	1500	3.23	3.80	
	Poor Control (PGC)	1407	3.96	4.54	<0.001
CRP (Exploratory)	Good Control	1503	29.52	59.19	
	Poor Control	1408	50.93	81.14	<0.001

**Table 3:** Independent samples *t*-test for NLR (primary) and CRP (exploratory) between glycemic control groups.

Marker	Levene’s Test F	Significance	t	df	Mean Difference	Standard Error Difference	95% CI (Lower–Upper)
NLR	26.20	<0.001	4.72	2905	0.73	0.15	0.43–1.03
CRP (Exploratory)	134.90	<0.001	8.17	2909	21.41	2.62	16.27–26.55

**Table 4:** NLR percentile analysis as an inflammatory marker percentile distribution (25th, 50th, 75th percentiles).

Marker	Group	25th	Median (50th)	75th
NLR	Good Glycemic Control	1.12	1.93	3.74
	Poor Glycemic Control	1.32	2.28	4.71
CRP	Good Glycemic Control	2.04	6.58	22.81
	Poor Glycemic Control	3.52	11.67	59.70

**Table 5:** ROC curve analysis.

Marker	AUC (Area Under Curve)	95% CI	*p*-Value
NLR	0.560	0.539–0.580	0.001
CRP	0.593	0.573–0.614	0.001

## Data Availability

The datasets generated and/or analyzed during the current study are not publicly available due to institutional and ethical restrictions involving patient confidentiality. However, de-identified data may be made available from the corresponding author upon reasonable request and with appropriate approvals from the institutional ethics committee.
